# Realising agency: insights from participatory research with learners in a South African sexual and reproductive health programme

**DOI:** 10.3389/fpubh.2024.1329425

**Published:** 2024-10-09

**Authors:** Chelsea Coakley, Devyn Lee, Carey Pike, Laura Myers, Miriam Hartmann, Asantewa Oduro, Noluthando Ntlapo, Linda-Gail Bekker, Youth Investigators of the Goals for Girls study

**Affiliations:** ^1^Desmond Tutu HIV Centre, Department of Medicine, Faculty of Health Sciences, University of Cape Town, Cape Town, South Africa; ^2^Centre for Social Science Research, Faculty of Humanities, University of Cape Town, Cape Town, South Africa; ^3^Grassroot Soccer, Cape Town, South Africa; ^4^Women’s Global Health Imperative, RTI, Berkeley, CA, United States; ^5^Department of Global Public Health, Karolinska Institutet, Stockholm, Sweden; ^6^Perinatal HIV Research Unit, Faculty of Health Sciences, University of the Witswatersrand, Johannesburg, South Africa; ^7^South African Medical Research Council, Cape Town, South Africa

**Keywords:** participatory research, youth & adolescence, intervention research, sexual and reproductive health, health promotion

## Abstract

**Background:**

Investing in the capabilities of adolescents is essential to achieving the United Nations Sustainable Development Goals, which focus on realising adolescent girls and young women’s (AGYW) rights to education, health, bodily autonomy and integrity, sexual and reproductive health (SRH) and well-being. Despite significant scientific and programmatic progress in understanding and responding to their unique and intersecting vulnerabilities, AGYW continue to face disproportionate risk of STIs, HIV and early pregnancy. Health promotion and preventative interventions stand to be improved by early and meaningful engagement of AGYW in intervention design and delivery.

**Methods:**

This study employed Youth Participatory Action Research (YPAR) to co-generate lessons for future school-based SRH programming. The 5-step YPAR process included: (1) youth investigator recruitment; (2) youth investigator training and co-design of YPAR methods; (3) youth investigator-led data collection; (4) collaborative analysis and interpretation; and (5) dissemination.

**Results:**

Collaborative analysis revealed improvements in self-concept and bodily autonomy, understanding and formation of healthy relationships and demand for girl-centred health services and information at school. Additionally, the study highlights YPAR’s positive influence on both the collaborative process and outputs of research. Further, it provides further insight into the quantitative biomedical and socio-behavioural findings of a larger experimental impact evaluation, in which it was nested.

**Conclusion:**

Results from YPAR methods point to high programme acceptability and practical lessons to inform future school-based SRH programming. The inclusion of adolescent girls in the design, delivery and evaluation of intervention research that affects their lives is an important strategy for improving acceptability, and also has demonstrated value in building their health and social assets. Future recommendations include parental involvement, and employing quantitative measures for better evaluation of youth engagement, leadership and partnerships in the research process.

## 1 Introduction

Investing in the capabilities of adolescents is essential to achieving the United Nation’s Sustainable Development Goals, which focus on realising adolescent girls and young women’s (AGYW) rights to education, health, bodily autonomy and integrity, sexual and reproductive health (SRH) and well-being (1, 2). AGYW continue to face disproportionate risk of sexually transmitted infections (STIs), HIV and early pregnancy, despite significant scientific and programmatic progress in understanding and responding to their unique and intersecting vulnerabilities as well as the developmental, biological, structural and behavioural risks they face (3). In South Africa, increasing rates of adolescent pregnancy, persistent and disproportionate HIV infections and the syndemic interactions between negative health outcomes amongst AGYW call for urgent, tailored interventions, which stand to be improved by early and meaningful engagement of AGYW in intervention design and delivery (4–7).

Comprehensive sexuality education (CSE) is a critical component of securing universal access to SRH and rights for all adolescents (8). School-based CSE programmes have demonstrated improvements in learners’ SRH, as indicated by increased HIV knowledge, delayed sexual debut, improved self-efficacy to engage in safer sex, and a reduction in unintended pregnancy, intimate partner violence and unsafe abortions (9–13). Since 2000, CSE has been included in South Africa’s (SA) secondary school curricula through the examinable subject “Life Orientation” (LO), which covers health content alongside civic responsibility, career planning, and other life skills. However, LO has not led to a demonstrated improvement in learners’ health outcomes or self-reported sexual risk behaviour and has faced several implementation challenges (14, 15). A recent study evaluating the efficacy of scripted LO lesson plans in two SA provinces found no differences between intervention and control groups on HIV knowledge and attitudes, condom use, diagnoses of STIs or pregnancy incidence (16). For significant and measurable impact, the design and implementation of CSE must consider the unique features of adolescence, the contextual factors that shape their access to and participation in CSE, and encourage the direct and systematic involvement of adolescents (17).

Adolescent engagement paradigms have been inconsistently conceptualisated, have limited empirical data, and as a result, there are limited adolescent-engaged acceptability studies, especially within the African region (18). While the current approaches predominantly incorporate programme participants’ views at the intervention development phase (19), a shift towards meaningfully engaging adolescents as agentive actors during the research process and ongoing refinement of programmes can improve stakeholder support, intervention content and delivery (20, 21). Youth participatory action research (YPAR) is an innovative, equity-focused, positive youth development approach that engages youth as experts and researchers and takes into consideration the developmental needs of adolescents (22). Participation in YPAR supports positive youth development by creating an opportunity for skill development, power-sharing and leadership. Young researchers are supported to inquire, navigate, reflect and act on their individual and shared experience, building collective agency and momentum in the process (23, 24). YPAR draws on young researchers’ valuable insider perspectives and lived experiences, stands to improve the validity of research findings, and offers an ethical and empowering platform to co-produce new knowledge and practical solutions in intervention development and evaluation (22).

Applying this concept, a YPAR sub-study was conceptualised and nested within a larger cluster-randomised controlled trial (cRCT) that sought to evaluate the impact of a sport-based, after-school SRH programme (called “SKILLZ”) on pregnancy, STIs, and socio-behavioural indicators amongst female learners aged 14–17 years in Cape Town, South Africa. This manuscript describes the in-depth youth participation action research methods used in a qualitative sub-study designed to explore (1) programme acceptability and impact on AGYW’s decision-making processes in relationships and (2) the factors that shaped their access to and experience of SRH information and services. Results highlight unique insights gained from this approach, along with youth investigator reflections on their perceived benefits (25).

## 2 Methods

### 2.1 Study setting and design

This sub-study took place in the Klipfontein and Mitchells Plains health sub-district of Cape Town, South Africa. This sub-district is a high-density, multi-disease-burdened, under-served peri-urban area (26). According to our baseline survey, in 2019 this area was predominantly isiXhosa speaking, then English, then Afrikaans. Unemployment is high (>50%), and even higher amongst young people aged 15–24, approximately 61% in the 2nd quarter of 2023 (27). The median age of sexual debut amongst South African adolescents overall is at 15.22 years (28). STI rates amongst adolescent girls are particularly high: as highlighted by STI monitoring in Pre-Exposure Prophylaxis (PrEP) demonstration projects in the Cape Town metro area, there was a very high prevalence of *Chlamydia trachomatis* (up to 40%) and Neisseria gonorrhoea (up to 11%) has been consistently found amongst AGYW in this region, with co-infection rates of up to 28% (29, 30). One third of women were pregnant during adolescence, with the last four years (2017–2021) seeing a staggering increase of 48.7% in births amongst 10–14 year-olds and 17.9% amongst 15–19 year-olds (31). Unintended pregnancies amongst learners in South Africa is a growing challenge, and a recent study in the Western Cape demonstrated that although the majority of adolescent mothers found that contraception was easy to access (61.1%) and readily available (76.4%), only 12.1% were using contraception at the time of conception and 76.4% felt their pregnancies had occurred at the “wrong time” (32).

#### 2.1.1 Overview of the cluster-randomised trial, goals for girls

Goals for Girls was a cRCT, conducted between 2018 and 2019, that evaluated a sport-based SRH programme called SKILLZ for female learners aged 14–17 years. The trial included 36 secondary schools (18 intervention schools; 18 control schools) within a single health sub-district in Cape Town, South Africa. Intervention schools received the SKILLZ programme alongside the national CSE programme, while control schools received only the national CSE programme. The trial sought to determine the impact of the SKILLZ programme on SRH outcomes, including STI prevalence and pregnancy incidence; the primary results of this investigation have been published elsewhere (25). Ethical approval for the trial and sub-study was granted by the Human Research Ethics Committee of University of the Cape Town (REC 137/2018). The trial was registered with BMC Trials ISRCTN77395422 https://www.isrctn.com/ISRCTN77395422.

#### 2.1.2 SKILLZ intervention

The SKILLZ curriculum was adapted from an existing SRH programme to align with and augment the national LO curriculum and respond to recent literature about the need to address gender and power in sexuality education (11, 33). The girl-centred, participatory SKILLZ curriculum explored gender attitudes, norms and power relations, healthy relationships, self-concept and self-efficacy and other SRH and rights themes. SKILLZ combined sport-based activities and facilitated discussion in 10 weekly sessions that were either delivered during or after school. Sessions were led by near-peer young female “Coaches” (<30 years old), non-professional group facilitators and role models of a similar age, gender, and social group to the learners. This approach was informed by evidence that consistent session exposure and strong strength facilitator-participant relationships impact SRH through increased HIV knowledge, awareness of local SRH services, and uptake of HIV testing (33, 34). SKILLZ was based on the premise that sport is a transformative platform to support the health and well-being of girls during a time of significant development, and has the potential to build girls’ health and social assets, create safe spaces for them to challenge and reconstruct traditional gender scripts, and support them in building new skills and confidence (20). Mentor/facilitator coaches are particularly important as the safe spaces they create and relationships they build with participants are highly valued (19, 21). In addition to the benefits of physical activity, sport-based activities provide adolescent girls with a platform to develop and expand social support networks, learn and apply new skills, and lead in a space that is traditionally male-dominated (34).

### 2.2 Overview of the YPAR sub-study

The accompanying YPAR sub-study aimed to add depth to parallel traditional qualitative methods, which included focus group discussions (FGDs) with participants with high and low programme attendance rates and in-depth interviews (IDIs) with school staff that aimed to describe the acceptability of the programme, assess the feasibility of implementing the programme, and to determine the individual factors associated with pregnancy and STI prevalence. We anticipated that a number of hypothesized intervening pathway concepts might prove difficult to quantitatively measure with sufficient precision in the survey, motivating for the use of both traditional and non-traditional qualitative methods to investigate acceptability of the integrated programme.

We engaged theories of positive youth development, framed within a socio-ecological model of health (35, 36) in the design of this sub-study that recognised adolescents as experts of their own lives, capable of expressing and capturing how complex individual, interpersonal and social factors shape their experiences and environment (37–39). The sub-study explored (1) learners’ social experiences at school; (2) SRH experiences, beliefs, values and behaviours; (3) perceptions of the SKILLZ programme; (4) interactions with SKILLZ Coaches, and (5) whether and how programme participation affected their behaviour and decision-making.

### 2.3 YPAR study process

**Figure 1:**
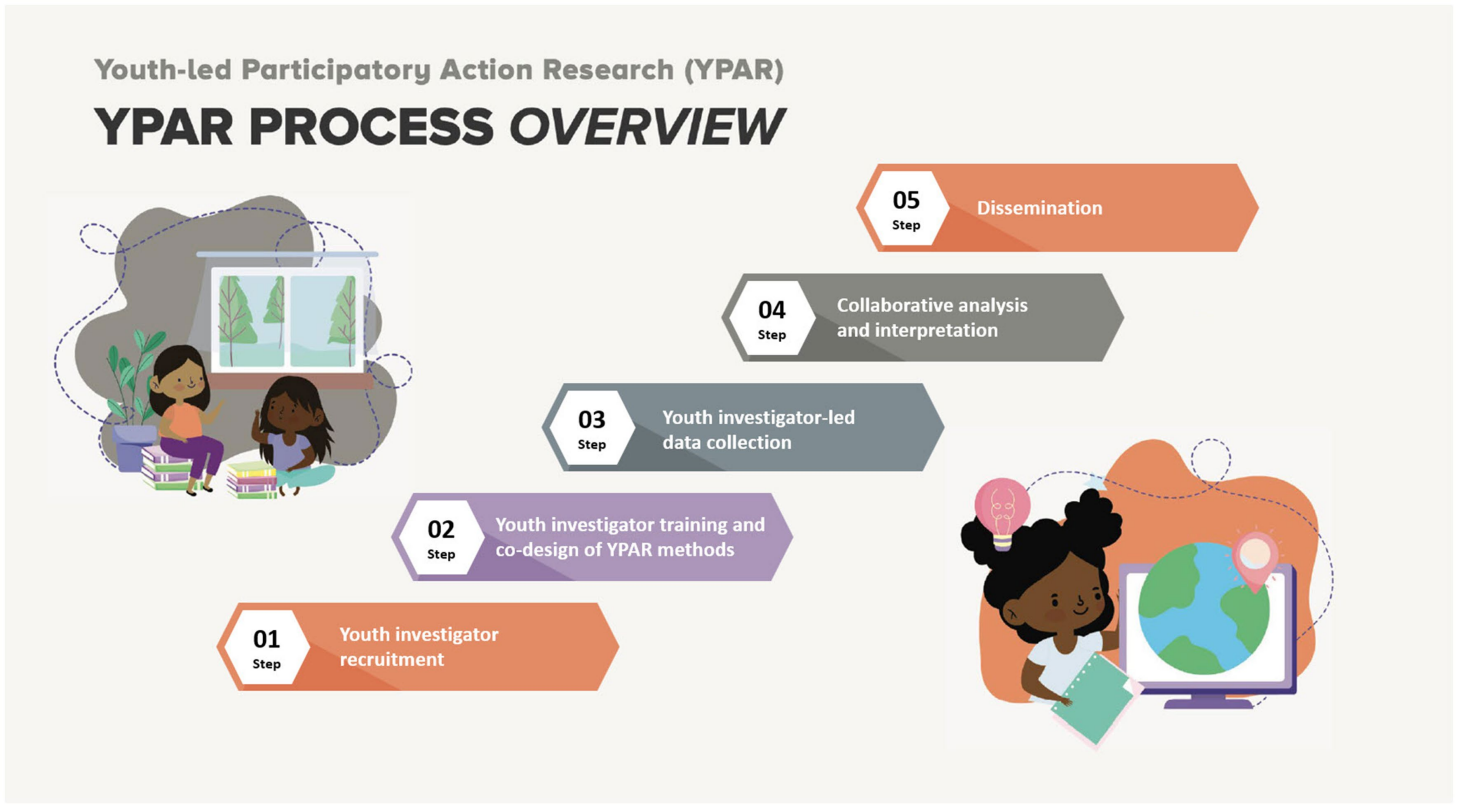
YPAR process overview.

This methodology included multiple steps, as summarized in Figure 1: (1) Youth investigator recruitment; (2) Youth investigator training and co-design of YPAR methods; (3) Youth investigator-led data collection; (4) Collaborative analysis and interpretation; (5) Dissemination.

#### 2.3.1 Youth investigator recruitment

Contact teachers from two intervention and two control schools nominated nine youth investigators (YIs), aged 14 to 17 years, that demonstrated programme interest, an ability to work well in a team, good communication and leadership skills, a sense of curiosity, and an interest in developing research and advocacy skills. The YI group (*n* = 9) had diverse characteristics (Table 1), including those with lower programme engagement, to ensure a broad range of perspectives and experiences was represented in YI-led methods. YIs agreed to participate as co-researchers, supported by experienced adult researchers and research assistants. YIs received a R100 voucher (equivalent to $14.50 US based on the 2019 average exchange rate) to acknowledge their time and safe transportation for each of the training sessions, data collection and analysis workshops they attended. Sessions also included refreshments, safe transport directly to their homes, and personalized certificates were provided to 5 YIs who completed the YPAR study.

**Table 1:** YPAR youth investigators’ demographic and SKILLZ participation characteristics.

YIs	Age (years)	Preferred language	School grade	SKILLZ sessions attended (attended / expected)	YPAR outcome
YI 1	14	English	9	3/10	Completed
YI 2	14	English	9	10/10*	Completed
YI 3	14	English	9	7/10*	Did not complete
YI 4	16	IsiXhosa	10	Control school	Completed
YI 5	16	IsiXhosa	10	Control school	Did not complete
YI 6	14	English	9	Control school	Complete
YI 7	14	Afrikaans	9	Control school	Did not complete
YI 8	14	IsiXhosa	8	6/10	Completed
YI 9	15	IsiXhosa	8	3/10	Did not complete

* Denotes “graduation” from the programme, or 70% attendance.

#### 2.3.2 Youth investigator training and co-design of YPAR methods

YIs were trained on research ethics, how to facilitate focus groups, ask open-ended questions and probe, take field notes, identify key messages, and categorise and analyse data. They were also coached on how to engage their peers and how to avoid dominating or directing participation. Between 2019 and 2020, pairs of YIs worked together with the study investigators and two Masters-level research assistants to plan and facilitate four YPAR workshops with their adolescent peers (*n* = 6–8 per school) during the cRCT. Preliminary research questions developed by the study team were discussed and refined with YIs, preparing them for data collection and analysis. The final research questions selected were: (i) *What factors influence whether girls get pregnant or an STI?;* (ii) *What could be done to create healthier gender norms in schools and relationships?*; (iii) *How do girls feel about SKILLZ?*; and (iv) *What are the most important parts of a dream [sexual and reproductive health] programme for girls?* Research questions guided co-development of workshop methodology and FGD guides. YIs reviewed, piloted and refined data collection tools with each other, supported by a pair of isiXhosa- and Afrikaans-speaking Research Assistants, in their preferred language. This exercise resulted in the removal of a visualisation exercise, simplification of the “dream programme” visual collage activity and expansion of the FGD guide to include new probes, which were developed by the YIs. In response to asking “what went well?” during the piloting of methods, they shared a range of perspectives.

#### 2.3.3 Young investigator-led data collection

Each pair of YIs facilitated a YPAR workshop for their school, supported by a pair of isiXhosa- and Afrikaans-speaking Research Assistants. YPAR workshop material contained multiple forms of physical, visual and recorded voice data, which were captured and translated into English and then transcribed by Research Assistants; participant names and other identifying information were not included in transcriptions. Following each YPAR workshop, a de-briefing meeting was held with YIs and Research Assistants to confer how the discussions went, how the facilitation could potentially be improved for the next session, and to reach consensus on the key findings and action points tobe shared with the programme team. These points were captured in process and observation notes, which were kept by the adult researchers and discussed with YIs at the conclusion of the study.

##### 2.3.3.1 Focus group discussions

YIs facilitated a focus group discussion as part of each YPAR workshop, supported by Research Assistants. FGD question guides included open-ended questions to elicit responses based on the factors affecting uptake, adherence and retention, followed by questions to probe the known factors affecting school and KGIS / SKILLZ attendance including age, education, and subjective and relational well-being.

##### 2.3.3.2 “Dream programme” activity

The “dream programme” activity engaged with visual participatory methodologies (VPM) and was used to conceptualise ideal programmatic components and design features. VPM was selected based on the extensive literature that posits that it can enhance meaningful engagement of young people, reduce power asymmetries, and provide an opportunity for young people to communicate complex feelings (37). Prior to the implementation of the YPAR sub-study, VPM had been recently used with urban, isiXhosa-speaking adolescents of similar age living in HIV-affected communities to consider implementation of healthcare interventions and services. In this study, VPM was established as a critical tool for expressing the multi-level factors that shape participation in and experience of health programming and consider the social ecology of adolescent lives (40).

**Figure 2:**
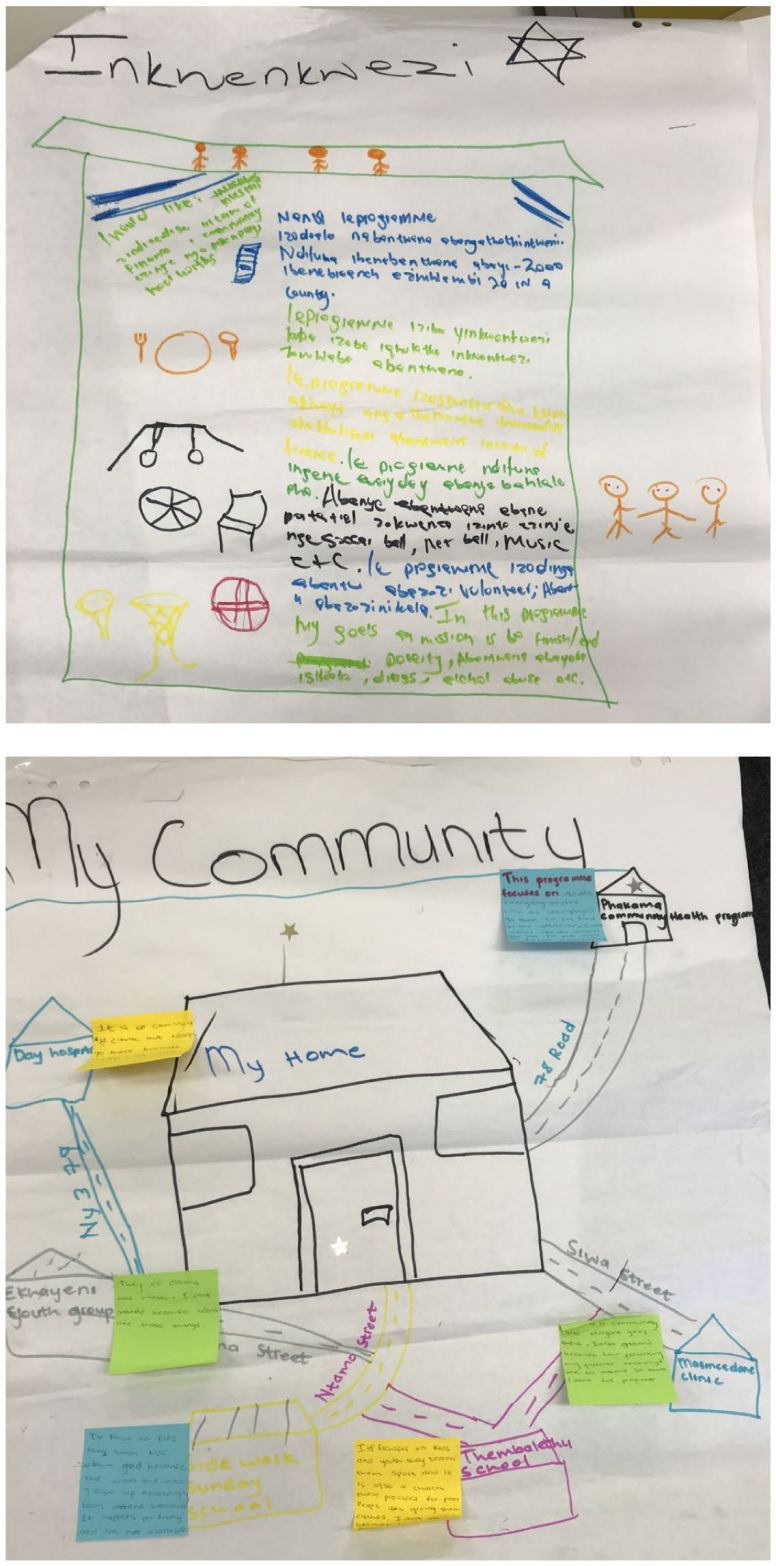
Images from arts-based dream programme activity.

In the YPAR substudy, YIs invited participants to discuss and design their dream youth CSE programme, critiquing current models of sexuality education at their schools and surrounding communities. They considered which elements were necessary to make programmes appealing, accessible, responsive and health-promoting. They also were encouraged to think about the existing design and delivery of sexual health services that are currently available to them and to visualise, draw and describe service components into their “dream programme,” imagining and documenting a place and/or experience different from what they already knew (Figure 2). Working alone or in small groups, participants were guided through a 3-step process by YIs, who supported them to: (1) express: their perceptions of health-promoting information, programmes and sexual health services, including perceived barriers to uptake; (2) share their experiences finding and navigating existing health programmes and facilities; and (3) draw the site(s), components and stakeholders that make up their ideal programme, discuss their drawings with the group, explaining the significance of what they included.

#### 2.3.4 Collaborative analysis and interpretation

##### 2.3.4.1 Collaborative analysis with YIs

The iterative analysis process included a debrief on the processes of data collection, field-note taking focused on their observations of and participation in the YPAR methods, participants, and themes emerging as well as a concluding collaborative analysis workshop, supported by RAs and supervised by the study’s co-investigators. The collaborative coding process included: (1) YIs reading through transcribed FGDs and interviews and summary observations, and reviewing images of completed collages; (2) YIs participating in an investigator-facilitated discussion guided by co-developed research questions and YI-selected excerpts; (3) YIs constructing codes and discussing arising themes.

##### 2.3.4.2 Thematic analysis and integration of YI analysis

Workshop discussions were recorded with key excerpts transcribed (e.g., when participants discuss their perceptions of health programmes and present their drawings). The interviewer and participant researchers took notes on what was discussed and noted significant points. YIs worked with research assistants to review their notes on what participants shared and drew, to reach consensus when summarising key findings.

A team of four analysts used Braun & Clarke’s six phases model (41, 42) for thematic analysis, Given that the participatory study set out to explore specific issues and concepts, but also aimed to discover unexpected aspects of the participants’ experience and co-produced knowledge, a combined deductive and inductive approach was used that was both critical and constructionist. Codes were extracted inductively, guided by the content of the data, and deductively, guided by the study’s conceptual framework and programme’s logic model. A preliminary codebook was developed based on topics covered in the SKILLZ curriculum and YPAR workshop guides. The codebook was applied to the subset of transcripts through open coding by two investigators, to determine the functioning and relevance of the codes. Revisions to the codebook were discussed and finalized amongst investigators, and used by three investigators to code the full dataset.

Coding was conducted using qualitative analysis software Dedoose version 8.0.35, a cloud application for managing, analysing, and presenting qualitative and mixed methods research data. After coding, the data was classified according to themes, and the resulting thematic structures were interpreted using theoretical and analytical concepts drawn from the study’s conceptual framework. Throughout the data analysis phase, team members held regular meetings to review progress with data analysis, to discuss and resolve issues and challenges arising from the analysis, and to ensure consistency in the use of the codebook.

Thematic interpretation focused on the acceptability and impact of the different programme models and the degree to which these influenced health behaviours, as well as the subjective “impact” of the programme on participants’ school, interpersonal, and health service experiences. After the thematic analysis process was completed, study investigators integrated themes through triangulation of data from YI-led collaborative coding, thematic discussions between YIs and adult investigators in a concluding analysis workshop, and adult-led coding and thematic analysis process that responded to the initial and YI-refined research methods. Process and observation notes were also considered and included as insights from the experience of YIs implementing the participatory study.

#### 2.3.5 Dissemination

A YI-led dissemination agenda and school-specific recommendations were prepared and planned for by YIs. However, due to strict COVID-19 lockdown restrictions in South Africa, this important face-to-face, YI-led portion of the YPAR study was not completed. Instead, a participatory, virtual dissemination event was convened in partnership with collaborating schools. The event sought to actively engage learners and educators from participating schools in a discussion around the results. Their feedback was summarised and published as a blog on the collaborating research organisations’s website. Due to strict COVID-19 lockdown restrictions, it was not possible to co-facilitate this event with YIs as intended; instead it was facilitated online by adult investigators.

## 3 Results

### 3.1 YPAR-generated data

#### 3.1.1 Adolescent participants in YPAR methods

Using purposive sampling that selected both high and low programme attenders, a subset of the overarching SKILLZ study participants were recruited from four schools (*n* = 30). Participants ranged from 13 and 17 years old, and were enrolled in grade 8–10 from two schools in the cRCT’s trial intervention arm and two schools in the control arm. Adolescent participants consented to participation in a half-day YPAR workshop at each school and, like YIs, were also given a R100 voucher, safe transportation, refreshments, and personalized certificates at YPAR study completion. With creative and in-depth contributions from these adolescent participants, youth investigators were able to collaboratively develop themes with support from our research team. These are detailed below and summarized within a socio-ecological model of health, engaged in the conceptualisation of this study (Table 2).

**Table 2:** YI-generated themes, mapped to domains of an ecological model-informed framework for building an enabling sexual and reproductive health environment for adolescents (35).

Framework domains	Research questions	YI-generated themes
Individual	What could be done to create healthier gender norms in schools and relationships?	Positive self-concept and bodily autonomy
Relationships		Formation of healthy relationships
Community	What are the most important parts of a dream programme for girls?	Girl-centred health services and information at school
Society		Formation of healthy relationships

#### 3.1.2 Positive self-concept and bodily autonomy

A critical finding, and one which forms the foundation for other programmatic impacts described, was an increase in self-esteem and confidence. As a result of participation in sessions, participants described a new sense of empowerment and bodily integrity, as well as body positivity:

“I learned to appreciate my body and have a positive self –talk about my body I must not criticize myself and I must not look down on me when I see other children wearing expensive clothes I learned that as long as I am not naked my body is covered there is no problem” (YPAR School 1).

“[SKILLZ] teaches us to say no and it also teaches us we have a say in who we trust with our bodies, and to respect our bodies and for people to respect our bodies also” (YPAR School 2).

Positive self-concept helped participants to navigate friendships and intimate relationships and plan to take action in their lives. For example, participants described how their new confidence allowed them to overcome pressures around sex and that they learned to value themselves more, instead of in relation to material things that other friends had, including from “blessors” (transactional, age-disparate, intimate relationships):

“[SKILLZ] taught us a lot of things; of how we need to put our status as a woman and a girl, and it also made us feel comfortable and confident in wanting to say no to sex” (YPAR School 2).

“I discovered my friends that are what they are and in October we were going to a Tour and my friends were telling me that they will have their own monies and boyfriends will buy for them so if I have little money I will have to use it otherwise they will not share theirs with me. This made me think of getting myself a boyfriend but I learned from [SKILLZ] that I can say no and I can change friends if I want to” (YPAR School 1).

“I also learned that when you do not want to do something and someone is forcing you to do it you can always say no you do not want to do it” (YPAR School 1).

#### 3.1.3 Formation of healthy relationships

The process of building a positive self-concept was closely connected to the value participants placed on the internal and external, social process of learning about, identifying and choosing healthy relationships, both in connection to friends and partners. Participants contrasted learning about topics in SKILLZ with the lack of those topics in the national curriculum, including healthy relationships, and how to realise individual agency and navigate friendships and intimacy with sexual partners. Participants further expressed a new understanding of abuse as being unhealthy.

“There was nothing like that from LO I did not know if the is abuse in your relationship that is called unhealthy relationship” (YPAR School 1).

“Don’t impress friends because some friends do not love you for who you, they just want to change who you are and be like them. So, just be yourself” (Adult-led FGD, School 5).

“First, you must focus on your books, because boys bring babies” (YPAR, School 1).

YPAR participants’ perspectives correlated well with feedback from SKILLZ Coaches regarding how participants’ understanding of healthy relationships, in terms of both intimate relationships and friendships. For example, one Coach highlighted the gaps in girls’ relationship knowledge prior to participating in SKILLZ, and how understanding all types of relationships is important:

“… they believe that it’s okay to be bullied by your boyfriend or girlfriend, it’s okay. Some of them, they don't know that it’s abuse … My favourite session was healthy and unhealthy relationships, because … it really doesn't focus on the partners, the relationship that you should have with your mum, with your brothers and sisters, and the relationship that they should have as mates, classmates…So it really built that bond with me and with them, that it’s really okay to have a good relationship within the class…” (Coach, post-intervention anonymous coach survey).

Expanding further on individual healthy relationships was an apparent enhanced ability to develop positive peer networks by addressing fears and mistrust that some participants carried around their peers, where they frequently framed peer relationships as threatening. Participants described being comfortable and open with their peers in the programme and learning how to ‘choose friends’ who will not pressure you to do things you do not want to do, like drink or have sex.

“I learned here if I want to start friendship, I must know how dangerous they are” (YPAR School 1).

“I took an action by choosing friends I saw the ones who were pretending me and wanted to destroy my life I got rid of them and many boyfriends as well I got rid of that because I learned from [SKILLZ] that behaviour is not good” (YPAR School 1).

Within SKILLZ, some participants felt that a more positive peer dynamic fostered a sense of collective agency and Coaches also reported growth in the SKILLZ group – observing a new sense of comradery amongst adolescent peer group members.

“They gave us a positive influence on who we really are and what we can actually do as a group, and they gave us a chance of knowing that we can work together as schools and as girls to fight for what we deserve” (YPAR School 2).

#### 3.1.4 Girl-centred health services and information at school

Traditional qualitative methods of data collection did not find that SKILLZ participants directly utilised health services as a result of participation, although some Coaches stated that participants told them about taking up health services. However, in YI-led focus group discussions, this topic was discussed and YIs probed participants to learn more about trusted sources of sexual health information. Participants expressed their desire to access nurses’ support and school-based girls groups for discussion and delivery of health information and services.

“[Principals must] let school have nurses that we can ask help from” (YPAR Collaborative Analysis Workshop).

“YI-2: How do you like to learn about sexual health?

P8: At the clinic from a nurse, because they know everything and will explain to you about what is safe to do and what is wrong.

YI-1: Like, do we all want to learn at the clinic?

P5: Groups like this for girls, maybe if we someone who knows better about sex who can explain to us, then we can share our opinions and teach each other. Like a girl’s organisation like Goals4Girls, there should be more at schools, they shouldn’t come once a month, so that every girl can have an understanding about sexual intercourse and what it comes with (YPAR, School 2).

F5: Problem is, at the clinic they cast you down. It is even hard to go there for contraceptives because the nurses cast you down. They have an attitude. Now if you had to ask her again on top of her being irritated all the time? We ask our friends. A friend that you know that has done this for a while so she’s experienced (YPAR, School 4).

As part of the overarching cRCT, health data was collected at two time points from study participants on their school’s premises, exposing them to the experience of mobile, on-site SRH services (such as HIV testing, STI testing, pregnancy testing, and SRH risk screening). This may have encouraged participants to see the benefit of such education and services and to start to advocate for them, as revealed in the YPAR group. However, the limited provision of and support for adolescent-responsive sexual and reproductive health services at schools in this region is a major limitation; this may also explain the discrepancy identified across methods (43).

The SKILLZ programme focused more specifically on health-related information compared to the national LO programme, which loosely combines health, career, and citizenship content.

“[Teachers] being scared to talk about sexual things keeps us from learning what good or wrong things we must know. Communicating with learners makes us feel free to ask questions. Do not judge us, you are there to guide us” (YPAR Collaborative Analysis Workshop).

“[SKILLZ] gave us more information than LO gave us. Goals4Girl[s] gave us information that only girls need to know and that made us feel good, it made us feel like we are someone, somewhere…” (YPAR School 2).

In the national CSE curriculum, SRH education is delivered to female and male learners simultaneously. SKILLZ participants, in contrast, expressed an appreciation for the delivery of SRH information in a girls-only space. In describing their visual depiction of the “dream programme” they described a girls’ group that provided multiple levels of support for health and well-being, which also reflects previously reported findings on the building of collective agency amongst group members.

“The difference between, they are different because in Life Orientation class, if the girls want to say something like, if they say they want to have a healthy relationship, boys will make fun of the girl and if you walk outside, they will tell their friends what you said” (YPAR School 2).

“It is far different because in LO class, many people talk at one time and their opinions is different because there is a male opinion of sexual and then there is a female opinion. In Goals4Girls, there is only one opinion, which is the female opinion” (YPAR School 2).

“So, guys my group is a girl’s support group…Times will be 16:00 because some of us finish school at 15:40, because when I get home I need to cook and clean, and then the programme will end at 18:00. It will take place on Mondays and Fridays. Why should you return? You will find support for the goals you have; people will share their experience…We will motivate each other, maybe if you have a child, you should not stop going to school. Some of us can’t open up to our mother because we are scared that they will shout at us, you will be able to open up at the girls support group. We will also warn others not to fall for the same mistakes; maybe you had a boyfriend at 14 and fell pregnant before 16, so we will guide you not to fall for the same mistake. If something happening at home is affecting you at school, we will also advise you. If someone is depressed and they have no help, we will help her find a solution, maybe speak to other people who were depressed” (YPAR School 2).

### 3.2 YI experiences and perspectives

Observation and process notes in combination with YI’s final written reflections demonstrated that the use of and participation in the process of YPAR was found to be acceptable to the YIs as well as to their peer participants. YIs described the experience of being a peer-researcher as “empowering” and that they “liked finding out more about what girls go through.” The majority of YIs appeared eager to participate and workshops were well-attended, with only two out of nine participants not completing the full process. YIs reported to find the training methods acceptable, although they reported a need for more extensive training when it came to facilitation skills. YIs found it challenging to probe participants to the degree that trained qualitative adult researcher may have done. A contribution from YIs during feedback and analysis workshops points to a possible result of this limitation:

“…they didn’t share everything it seemed like they held back; most of them want to learn more so they can expand knowledge and teach to others; everyone wants to learn about sex! You learn a lot more with girls, you get more comfortable and share a lot more” (YI, collaborative analysis workshop).

“…they weren’t as open as we expected and didn’t share much. They were quiet, we had to drag answers out of them”(YI, collaborative analysis workshop).

However, the results presented herein reflected that YIs were equipped and able to explore topics of interest to them and may have made their peers feel more comfortable in the interview process, thus enabling more authentic data collection.

Their contribution to interpretation of findings and dissemination was limited by time constraints and the implementation of COVID-19 lockdown restrictions in South Africa.

## 4 Discussion

This study successfully employed a YPAR approach to augment qualitative data collection from SKILLZ participants. YPAR was firstly found to be acceptable to both the YIs and their participant peers and secondly, it was capable of generating high-quality research data that enriched understanding of the impact of SKILLZ.

### 4.1 YPAR directly benefited youth investigators

YIs found the YPAR process interesting, engaging, and beneficial to them as individuals. The use of YPAR in other settings has resulted in significant personal benefits for YIs, whereby they were found to have a similarly enhanced capacity and space to reflect and engage on the content they were investigating (44). The YIs consistently held a positive view of the SKILLZ programme; a view that may have been influenced by their experience and involvement as leaders of the YPAR sub-study. A recent systematic review of acceptability research with adolescents whereby the early inclusion of adolescents at the beginning and throughout the intervention process was found to positively influence programme acceptability (18).

In SKILLZ, YIs were recruited from existing participants who were known to be engaged in the programme but had varying levels of attendance. Similarly, in other YPAR studies, more vulnerable adolescent representatives were recruited, which led to improved outcomes for YIs and the vulnerable group they represented: YIs recruited from low-performing student pools in a US study went on to be nominated and supported for scholarships, to become mentors for current students, and to advocate for enhanced support for other low-performing students (44). SKILLZ may have benefited from engaging a mix of high and low attending participants as YIs, who then may have been better positioned to represent the full range of participant experiences (44).

### 4.2 The evaluation of the SKILLZ programme benefited from the use of YPAR

YPAR findings confirmed that the SKILLZ intervention was highly valued amongst participants, affirming that school-based interventions focused on female secondary school learners should be responsive to external constraints, multi-level influences, and opportunities in adolescent girls’ lives – especially as their time and mobility is shaped by social and gender norms (45). Similar visual and narrative-based participatory methodologies have been documented as novel ways to engage with complexity in adolescents’ lives and have enhanced intervention design (46). Findings from the YI-led activities around the impact of SKILLZ are consistent with results from previous studies of the SKILLZ programme that used traditional, adult-led qualitative methods. SKILLZ participants in other studies reported improved self-concept, bodily autonomy, and feelings of empowerment, as well as improved confidence to make healthy decisions and stand up for themselves, attributing their improved confidence to the safe space created by the Coach and fellow participants (33, 34). A previous study of SKILLZ in South Africa echoed findings from the YI-led activities about improved confidence to take action and make decisions, where girls described strategies to exit abusive relationships while navigating the complexity of realising and executing their own agency (47). These findings overall are supported by the extensive literature on the disproportionate and intersecting vulnerabilities and SRH risks faced by adolescent girls, and the empirically-supported guidance that both research and programme design should engage with the social ecology of girls’ lives as much as possible, including their caregivers, relationships, school and health systems (35).

The facilitation skills and positive role-modeling of the near-peer Coaches have been highlighted as key components of SKILLZ that foster social–emotional growth amongst participants, consistent with a review of mentorship interventions that found they were associated with improved participant self-esteem, self-efficacy, and strengthened and increased peer networks (47, 48). Improved peer support has been reported as a positive effect of other facilitated group interventions for adolescents, where emotional support and trust in particular are connected to healthy coping skills and self-esteem (49, 50). A review of community-based girl group programmes found that they generally have positive effects on outcomes at the individual level that are independent of external factors, such as self-esteem, compared to those that also rely on external factors, such as utilisation of health services (51). This is consistent with findings from the YI-led activities where SKILLZ participants did not directly describe increased uptake of health services. An impact evaluation of PEPFAR-funding “DREAMS” (Determined, Resilient, Empowered, AIDS-free, Mentored and Safe) programming in Kenya and South Africa found that DREAMS beneficiaries reported higher social support across diverse implementation settings, and there was some evidence of improved self-efficacy amongst beneficiaries (52). Social asset-building approaches were specifically included to strengthen social connections of AGYW amongst peers and with DREAMS mentors; this is a similar approach to the SKILLZ intervention, which also aims to build assets of adolescent participants with the intention of being protective and promote access to health services and information (34, 52).

The YI-led participatory sub-study highlighted many themes, supported by documented implementation lessons from this study (53). Time constraints at the school level were an additional level on top of individual time commitments: for example, individuals have competing time for educational activities, home chores, other extra-mural activities, while at the school level there is competition for time in school partially because there is limited capacity to shift activities into after-school hours because of security risks.

### 4.3 Limitations and future research

The limitations of this study were found to be consistent with those described by others and may reflect the inherent limitations of this type of research (54, 55). Firstly, the positions of power and diverse identities of researchers and YIs likely influenced the research context, processes, and outcomes. For example, training and collaborative analysis sessions were facilitated mainly in English and although isiXhosa-speaking research assistants were trained on methodology and participated actively in the workshop and provided iterative translation, this may have constrained self-expression amongst first language isiXhosa and Afrikaans-speaking YIs. This challenge was mitigated in YI-led data collection where visual methodologies and FGDs were predominantly conducted in preferred language; participants were able to speak, probe and discuss amongst themselves in the language of their choice. Future collaborative research should ensure adequate resources and measures to ensure all stages of research are undertaken in the primary languages of YIs to ensure comfortable and full expression. Secondly, YIs were engaged mid-way through the cRCT study timeline and although the protocol and SKILLZ curriculum were reviewed and actively informed by a pre-existing Youth Advisory Group at this trial site, it would have been beneficial to recruit and engage the YIs earlier, e.g., from the conceptualisation phase, allowing them to co-develop initial research questions for the cRCT, themes for investigation and formulate and/or provide input on complementary qualitative methods and overall study design. Thirdly, teacher representatives from each school selected YIs which likely resulted in a degree of selection bias. Beyond SKILLZ programme attendance (see Table 1), additional individual-level data on the YIs was not collected, so it was not possible to report on the representativeness of this purposive cohort of YIs relative to their peers in the larger study. It is also worth noting that due to their dual role, as both participant (SKILLZ study) and investigator (YPAR sub-study), the YIs reported that, at times, they found it challenging to separate conclusions from their personal SKILLZ cRCT participant experience from the YPAR data analysis. They portrayed a strong desire to share their own experiences from the programme and while in some cases this emphasised and correlated strongly with YPAR findings, in other areas it may have overshadowed their ability to be objective. We propose building in further awareness of and allocating resources towards research objectivity, reflexivity and analysis skills as a more routinely revisited training and discussion area for YIs, in future collaborative research.

The lack of quantitative, systematic measures to evaluate the level of youth engagement in youth-adult partnerships is a further limitation. Future collaborative research between adults and youth should consider application of the validated Youth-Adult Partnership scale, which includes two sub-scales on Youth Voice in Decision-Making and Supportive Adult Partnerships (56). Combined with qualitative data, observations and process notes, this quantitative data would provide youth-adult research partnerships with more rigorous empirical evidence on the experience of youth in partnerships fraught with complex, age- and positionality-related power dynamics. Case study methodology or most significant change technique could be applied for investigation into the longer-term impact of this collaborative, interventional YPAR approach on YIs would be beneficial and follow-up should be considered in future research.

Finally, recent literature summarising research that was ongoing during this study’s conceptualisation and completion has demonstrated that health promotion interventions that address gender and power targeting adolescents require more multi-sectoral, multi-level engagement, where caregivers should also be considered as co-designers (57). Caregivers were not respondents in the current trial nor were they engaged in the participatory study design, however, their role as caregivers is an important consideration for future intervention design and participatory research.

## 5 Conclusion

While this participatory study demonstrated high levels of acceptability and reported impact of the SKILLZ programme amongst adolescent female learners, this did not translate to consistent attendance in the programme and did not impact implementation support at the school level (25, 53). In the cRCT, a wide variety of barriers to optimal implementation were observed across schools alongside modest impact on biomedical and socio-behavioural outcomes (25). Our research points to considerable scope for conceptualising and implementing future school-based participatory research methods in partnership with adolescent learners.

Our findings reinforce the literature on acceptability research with adolescents, where inclusion of adolescents earlier and throughout the intervention process positively influences acceptability. In the context of sexual and reproductive health, we recommend use of girl-centred, participatory design principles at the outset and throughout implementation and research activities. The inclusion of adolescent girls in the design, delivery and evaluation of intervention research that affects their lives is an important strategy for improving acceptability, and also has demonstrated value in building their health and social assets. YIs explored topics of interest to them and were able to create comfortability with their peers while conducting research. We recommend future studies consider YPAR as a complementary, positive youth development approach that complements and improves interpretation of findings from traditional, adult-let research methods.

## Data availability statement

The raw data supporting the conclusions of this article will be made available by the authors upon reasonable request and with permission of the data owners.

## Ethics statement

The studies involving humans were approved by University of Cape Town Faculty of Health Sciences Human Research Ethics Committee (HREC). The studies were conducted in accordance with the local legislation and institutional requirements. The ethics committee/institutional review board waived the requirement of written informed consent for participation from the participants or the participants’ legal guardians/next of kin because the study team, in consultation with the adult and youth Community Advisory Board (CAB) in this area proposed that this project is carried out with participants giving written informed consent prior to participation and involving their legal guardians or parents only if desired. The protocol team thus requested a parental proxy consent waiver with the following rationale: 1. Some participants might not feel comfortable talking to their parents/guardians about sexual and reproductive health (SRH) and parents/guardians may prevent participation in the programme due to the SRH content. 2. This is particularly the case with adolescents that find it the most difficult to talk to their caregivers about SRH, as they are often the ones at highest risk and that stand to benefit the most. 3. The high rates of HIV/STI infection and unintended pregnancy amongst this population make it that behavioural interventions are highly necessary and a public health priority, and SRH content is currently deployed through the national Department of Education without parental consent. 4. All the procedures offered (STI, HIV and pregnancy testing) in this protocol were carried out by trained, adolescent friendly staff and are all legal without parental consent in RSA above the age of 12 years. Any participant testing positive for HIV, STI or pregnancy were carefully counselled and appropriately treated or referred for treatment. Where possible, youth were encouraged to take a significant adult (or their parents) into their confidence as soon as they were comfortable to do so but within a 3 month period.

## Author contributions

CC: Conceptualization, Data curation, Investigation, Methodology, Writing – original draft, Writing – review & editing, Formal analysis, Funding acquisition, Project administration. DL: Data curation, Formal analysis, Investigation, Writing – original draft, Writing – review & editing. CP: Formal analysis, Funding acquisition, Methodology, Project administration, Writing – original draft, Writing – review & editing. LM: Conceptualization, Data curation, Formal analysis, Investigation, Methodology, Writing – review & editing. MH: Formal analysis, Writing – review & editing. AO: Project administration, Investigation, Writing – review & editing. NN: Project administration, Investigation, Writing – review & editing. L-GB: Funding acquisition, Investigation, Supervision, Writing – review & editing.

## References

[1] Sheehan P Sweeny K Rasmussen B Wils A Friedman HS Mahon J. Building the foundations for sustainable development: a case for global investment in the capabilities of adolescents. Lancet. (2017) 390:1792–806. doi: 10.1016/S0140-6736(17)30872-3, PMID: 28433259

[2] Gugliemi S Neumeister E Jones N. Adolescents, youth and the SDGs: What can we learn from the current data? London: Gender and Adolescence: Global Evidence (2021).

[3] UNAIDS. IN DANGER: UNAIDS global AIDS update 2022. Geneva: Joint United Nations Programme on HIV/AIDS (2022) Available at: https://www.unaids.org/sites/default/files/media_asset/2022-global-aids-update_en.pdf.

[4] Carbone NB Njala J Jackson DJ Eliya MT Chilangwa C Tseka J. “I would love if there was a young woman to encourage us, to ease our anxiety which we would have if we were alone”: adapting the Mothers2Mothers Mentor mother model for adolescent mothers living with HIV in Malawi. PLoS One. (2019) 14:e0217693. doi: 10.1371/journal.pone.0217693, PMID: 31173601 PMC6555548

[5] Mhungu A Sixsmith J Burnett E. Adolescent girls and young Women’s experiences of living with HIV in the context of patriarchal culture in sub-Saharan Africa: a scoping review. AIDS Behav. (2023) 27:1365–79. doi: 10.1007/s10461-022-03872-6, PMID: 36318422 PMC10129999

[6] Murewanhema G Musuka G Moyo P Moyo E Dzinamarira T. HIV and adolescent girls and young women in sub-Saharan Africa: a call for expedited action to reduce new infections. IJID Reg. (2022) 5:30–2. doi: 10.1016/j.ijregi.2022.08.009, PMID: 36147901 PMC9485902

[7] Duby Z McClinton Appollis T Jonas K Maruping K Dietrich J LoVette A. “As a young pregnant girl… the challenges you face”: exploring the intersection between mental health and sexual and reproductive health amongst adolescent girls and young women in South Africa. AIDS Behav. (2021) 25:344–53. doi: 10.1007/s10461-020-02974-3, PMID: 32683636 PMC7368608

[8] Germain A Sen G Garcia-Moreno C Shankar M. Advancing sexual and reproductive health and rights in low- and middle-income countries: implications for the post-2015 global development agenda. Glob Public Health. (2015) 10:137–48. doi: 10.1080/17441692.2014.986177, PMID: 25628182 PMC4318089

[9] Amaugo LG Papadopoulos C Ochieng BMN Ali N. The effectiveness of HIV/AIDS school-based sexual health education programmes in Nigeria: a systematic review. Health Educ Res. (2014) 29:633–48. doi: 10.1093/her/cyu002, PMID: 24572458

[10] Chavula MP Svanemyr J Zulu JM Sandøy IF. Experiences of teachers and community health workers implementing sexuality and life skills education in youth clubs in Zambia. Glob Public Health. (2022) 17:926–40. doi: 10.1080/17441692.2021.1893371, PMID: 33661081

[11] Haberland NA. The case for addressing gender and power in sexuality and HIV education: a comprehensive review of evaluation studies. Int Perspect Sex Reprod Health. (2015) 41:31. doi: 10.1363/4103115 25856235

[12] Fonner VA Armstrong KS Kennedy CE O’Reilly KR Sweat MD. School based sex education and HIV prevention in low- and middle-income countries: a systematic review and Meta-analysis. PLoS One. (2014) 9:e89692. doi: 10.1371/journal.pone.0089692, PMID: 24594648 PMC3942389

[13] Vanwesenbeeck I Westeneng J De Boer T Reinders J Van Zorge R. Lessons learned from a decade implementing comprehensive sexuality education in resource poor settings: *the world starts with me*. Sex Educ. (2016) 16:471–86. doi: 10.1080/14681811.2015.1111203

[14] Visser MJ. Life skills training as HIV/AIDS preventive strategy in secondary schools: evaluation of a large-scale implementation process. SAHARA J. (2005) 2:203–16. doi: 10.1080/17290376.2005.9724843, PMID: 17601024

[15] Francis DA DePalma R. “You need to have some guts to teach”: teacher preparation and characteristics for the teaching of sexuality and HIV/AIDS education in south African schools. SAHARA J. (2015) 12:30–8. doi: 10.1080/17290376.2015.1085892, PMID: 26365812

[16] Speizer IS Mandal M Xiong K Makina N Hattori A Durno D. Impact evaluation of scripted lesson plans for HIV-related content in a life orientation curriculum: results from two provinces in South Africa. BMC Public Health. (2020) 20:1542. doi: 10.1186/s12889-020-09640-2, PMID: 33054742 PMC7556937

[17] UNESCO, Joint United Nations Programme on HIV/AIDS, United Nations Children’s Fund, United Nations Entity for Gender Equality and the Empowerment of Women, World Health Organization. International technical guidance on sexuality education: An evidence-informed approach UNESCO (2018) Available at: https://unesdoc.unesco.org/ark:/48223/pf0000260770.

[18] Somefun OD Casale M Haupt Ronnie G Desmond C Cluver L Sherr L. Decade of research into the acceptability of interventions aimed at improving adolescent and youth health and social outcomes in Africa: a systematic review and evidence map. BMJ Open. (2021) 11:e055160. doi: 10.1136/bmjopen-2021-055160, PMID: 34930743 PMC8689197

[19] Bazzano AN Martin J Hicks E Faughnan M Murphy L. Human-centred design in global health: a scoping review of applications and contexts. PLoS One. (2017) 12:e0186744. doi: 10.1371/journal.pone.0186744, PMID: 29091935 PMC5665524

[20] Mannell J Willan S Shahmanesh M Seeley J Sherr L Gibbs A. Why interventions to prevent intimate partner violence and HIV have failed young women in southern Africa. J Int AIDS Soc. (2019) 22:e25380. doi: 10.1002/jia2.25380, PMID: 31441229 PMC6706780

[21] Casale M Yates R Gittings L Haupt Ronnie G Somefun O Desmond C. Consolidate, conceptualize, contextualise: key learnings for future intervention acceptability research with young people in Africa. Psychol Health Med. (2022) 27:181–92. doi: 10.1080/13548506.2022.2108078, PMID: 35938622 PMC10029093

[22] Ozer EJ. Youth-led participatory action research: overview and potential for enhancing adolescent development. Child Dev Perspect. (2017) 11:173–7. doi: 10.1111/cdep.12228

[23] Ozer EJ Douglas L. The impact of participatory research on urban teens: an experimental evaluation. Am J Community Psychol. (2013) 51:66–75. doi: 10.1007/s10464-012-9546-2 22875686

[24] Ozer EJ Abraczinskas M Duarte C Mathur R Ballard PJ Gibbs L. Youth participatory approaches and health equity: Conceptualization and integrative review. Am J Community Psychol. (2020) 66:267–278. doi: 10.1002/ajcp.12451 32969506

[25] Pike C Coakley C Ahmed N Lee D Little F Padian N. Goals for girls: a cluster-randomized trial to investigate a school-based sexual health programme amongst female learners in South Africa. Health Educ Res. (2023) 38:375–91. doi: 10.1093/her/cyad025, PMID: 37405698 PMC10516375

[26] National Department of Health, Statistics South Africa, South African Medical Research Council, ICF. South Africa demographic and health survey 2016 key findings. Pretoria, South Africa and Rockville, Maryland: NDoH, Stats SA, SAMRC, and ICF (2018).

[27] Statistics South Africa. Quarterly labour force survey 2023 Q2: statistical release P0211 Statistics South Africa (2023) Available at: www.statssa.gov.za.

[28] Layland EK Ram N Caldwell LL Smith EA Wegner L. Leisure boredom, timing of sexual debut, and co-occurring behaviors among south African adolescents. Arch Sex Behav. (2021) 50:2383–94. doi: 10.1007/s10508-021-02014-8, PMID: 34401994 PMC8911384

[29] Gill K Celum C Breen G Thomas K Morton J Baeten J. P432 high prevalence and incidence of curable STIs among young women initiating PrEP in a township in South Africa In: Poster presentations: BMJ Publishing Group Ltd (2019). A205.2–A205. Available at: https://sti.bmj.com/lookup/doi/10.1136/sextrans-2019-sti.518

[30] Delany-Moretlwe S Mgodi N Bekker LG Baeten JM Li C Donnell D. High prevalence and incidence of gonorrhoea and chlamydia in young women eligible for HIV pre-exposure prophylaxis in South Africa and Zimbabwe: results from the HPTN 082 trial. Sex Transm Infect. (2023) 99:433–9. doi: 10.1136/sextrans-2022-055696, PMID: 36889914 PMC10555488

[31] Barron P Subedar H Letsoko M Makua M Pillay Y. Teenage births and pregnancies in South Africa, 2017–2021 – a reflection of a troubled country: analysis of public sector data. S Afr J Obstet Gynaecol (1999). (2022) 112:252–8. doi: 10.7196/SAMJ.2022.v112i4.16327, PMID: 35587803

[32] Vollmer LR Van Der Spuy ZM. Contraception usage and timing of pregnancy among pregnant teenagers in Cape Town, South Africa. Int J Gynecol Obstet. (2016) 133:334–7. doi: 10.1016/j.ijgo.2015.10.011 26895740

[33] Merrill KG Merrill JC Hershow RB Barkley C Rakosa B DeCelles J. Linking at-risk south African girls to sexual violence and reproductive health services: a mixed-methods assessment of a soccer-based HIV prevention program and pilot SMS campaign. Eval Program Plann. (2018) 70:12–24. doi: 10.1016/j.evalprogplan.2018.04.010, PMID: 29890449 PMC6613633

[34] Hershow R Gannett K Merrill J Kaufman BE Barkley C DeCelles J. Using soccer to build confidence and increase HCT uptake among adolescent girls: a mixed-methods study of an HIV prevention programme in South Africa. Sport Soc. (2015) 18:1009–22. doi: 10.1080/17430437.2014.997586, PMID: 26997967 PMC4795818

[35] Svanemyr J Amin A Robles OJ Greene ME. Creating an enabling environment for adolescent sexual and reproductive health: a framework and promising approaches. J Adolesc Health. (2015) 56:S7–S14. doi: 10.1016/j.jadohealth.2014.09.011, PMID: 25528980

[36] Catalano RF Skinner ML Alvarado G Kapungu C Reavley N Patton GC. Positive youth development programs in low- and middle-income countries: a conceptual framework and systematic review of efficacy. J Adolesc Health. (2019) 65:15–31. doi: 10.1016/j.jadohealth.2019.01.024, PMID: 31010725

[37] Brown A Spencer R McIsaac JL Howard V. Drawing out their stories: a scoping review of participatory visual research methods with newcomer children. Int J Qual Methods. (2020) 19:160940692093339. doi: 10.1177/1609406920933394

[38] Montreuil M Carnevale FA. A concept analysis of children’s agency within the health literature. J Child Health Care. (2016) 20:503–11. doi: 10.1177/1367493515620914 26666263

[39] Cahill H Dadvand B. Re-conceptualising youth participation: a framework to inform action. Child Youth Serv Rev. (2018) 95:243–53. doi: 10.1016/j.childyouth.2018.11.001

[40] Hodes R Doubt J Toska E Vale B Zungu N Cluver L. The stuff that dreams are made of: HIV-positive adolescents’ aspirations for development. J Int AIDS Soc. (2018) 21:e25057. doi: 10.1002/jia2.25057, PMID: 29485764 PMC5978641

[41] Braun V Clarke V. Using thematic analysis in psychology. Qual Res Psychol. (2006) 3:77–101. doi: 10.1191/1478088706qp063oa

[42] Braun V Clarke V. Thematic analysis In: Cooper H Camic PM Long DL Panter AT Rindskopf D Sher KJ, editors. APA handbook of research methods in psychology, Vol 2: Research designs: quantitative, qualitative, neuropsychological, and biological. Washington: American Psychological Association (2012). 57–71. Available at: http://content.apa.org/books/13620-004

[43] Ahmed N Pike C Lee J Wagner C Bekker LG. School-based healthcare services in Cape Town, South Africa: when there’s a will, there’s a way. Afr J Prim Health Care Fam Med. (2023) 15:e1–3. doi: 10.4102/phcfm.v15i1.4216 PMC10623473 37916715

[44] Lárez NA Sharkey JD Frattaroli S Avila E Medina A. Implementing youth participatory action research at a continuation high school. Health Serv Res. (2023) 58:198–206. doi: 10.1111/1475-6773.14190, PMID: 37282759 PMC10339165

[45] Blum RW Mmari K Moreau C. It begins at 10: how gender expectations shape early adolescence around the world. J Adolesc Health. (2017) 61:S3–4. doi: 10.1016/j.jadohealth.2017.07.009, PMID: 28915989 PMC5612023

[46] Boehmer E Desmond C Mahali A Musarurwa H. Storying ourselves: black consciousness thought and adolescent agency in 21st-century Africa. J Postcolon Writ. (2021) 57:794–811. doi: 10.1080/17449855.2021.1954542

[47] Moolman B Essop R Tolla T. Navigating agency: adolescents’ challenging dating violence towards gender equitable relationships in a south African township. S Afr J Psychol. (2020) 50:540–52. doi: 10.1177/0081246320934363

[48] Plourde KF Ippoliti NB Nanda G McCarraher DR. Mentoring interventions and the impact of protective assets on the reproductive health of adolescent girls and young women. J Adolesc Health. (2017) 61:131–9. doi: 10.1016/j.jadohealth.2017.03.002 28528208

[49] Duby Z Verwoerd W McClinton Appollis T Jonas K Maruping K Dietrich JJ. “In this place we have found sisterhood”: perceptions of how participating in a peer-group club intervention benefited south African adolescent girls and young women. Int J Adolesc Youth. (2021) 26:127–42. doi: 10.1080/02673843.2021.1898423

[50] Arndt N Naudé L. Responsibility in the face of adversity: adolescents’ sense of self in reciprocal relationships. Youth Soc. (2020) 52:288–307. doi: 10.1177/0044118X17743992

[51] Temin M Heck CJ. Close to home: evidence on the impact of community-based girl groups. Glob Health Sci Pract. (2020) 8:300–24. doi: 10.9745/GHSP-D-20-00015, PMID: 32606096 PMC7326521

[52] Gourlay AJ Birdthistle I Mulwa S Mthiyane NT Magut F Chimbindi N. Awareness and uptake of the determined, resilient, empowered, AIDS-free, mentored and safe HIV prevention package over time among population-based cohorts of young women in Kenya and South Africa. AIDS. (2022) 36:S27–38. doi: 10.1097/QAD.0000000000003120, PMID: 35766573

[53] Pike C Coakley C Lee D Daniels D Ahmed N Hartmann M. Lessons learned from the implementation of a school-based sexual health education program for adolescent girls in Cape Town, South Africa. Glob Health Sci Pract. (2023) 11:e2300026. doi: 10.9745/GHSP-D-23-00026, PMID: 38124019 PMC10749656

[54] Wilhelm AK Pergament S Cavin A Bates N Hang M Ortega LE. Lessons learned in implementing youth and parent participatory action research in a school-based intervention. Prog Community Health Partnersh. (2021) 15:15–36. doi: 10.1353/cpr.2021.0002, PMID: 33775958 PMC9014976

[55] Kim J. Youth involvement in participatory action research (PAR). Crit Soc Work. (2019) 17:38–52. doi: 10.22329/csw.v17i1.5891

[56] Zeldin S Krauss SE Collura J Lucchesi M Sulaiman AH. Conceptualizing and measuring youth–adult Partnership in Community Programs: a cross National Study. Am J Community Psychol. (2014) 54:337–47. doi: 10.1007/s10464-014-9676-9, PMID: 25216734

[57] Mmari K Gayles J Lundgren R Barker K Austrian K Levtov R. Implementing interventions to address gender and power inequalities in early adolescence: utilizing a theory of change to assess conditions for success. J Adolesc Health. (2023) 73:S5–S14. doi: 10.1016/j.jadohealth.2022.10.032, PMID: 37330821

